# Whole-Genome Sequencing Shows That Patient-to-Patient Transmission Rarely Accounts for Acquisition of *Staphylococcus aureus* in an Intensive Care Unit

**DOI:** 10.1093/cid/cit807

**Published:** 2013-12-12

**Authors:** James R. Price, Tanya Golubchik, Kevin Cole, Daniel J. Wilson, Derrick W. Crook, Guy E. Thwaites, Rory Bowden, A. Sarah Walker, Timothy E. A. Peto, John Paul, Martin J. Llewelyn

**Affiliations:** 1 Department of Microbiology and Infection, Royal Sussex County Hospital, Brighton; 2 Department of Statistics, University of Oxford; 3 Public Health England, Royal Sussex County Hospital, Brighton; 4 Nuffield Department of Clinical Medicine, Experimental Medicine Division, John Radcliffe Hospital; 5 Wellcome Trust Centre for Human Genetics, Roosevelt Drive; 6 NIHR Oxford Biomedical Research Centre, John Radcliffe Hospital, Oxford; 7 Centre for Clinical Infection and Diagnostics Research, Guy's and St Thomas’ Hospitals NHS Foundation Trust, London;; 8 Division of Medicine, Brighton and Sussex Medical School, Falmer, United Kingdom

**Keywords:** *Staphylococcus aureus* transmission, whole-genome sequencing, *spa* typing, intensive care unit, adult

## Abstract

***Background.*** Strategies to prevent *Staphylococcus aureus* infection in hospitals focus on patient-to-patient transmission. We used whole-genome sequencing to investigate the role of colonized patients as the source of new *S. aureus* acquisitions, and the reliability of identifying patient-to-patient transmission using the conventional approach of *spa* typing and overlapping patient stay.

***Methods.*** Over 14 months, all unselected patients admitted to an adult intensive care unit (ICU) were serially screened for *S. aureus*. All available isolates (n = 275) were *spa* typed and underwent whole-genome sequencing to investigate their relatedness at high resolution.

***Results.*** *Staphylococcus aureus* was carried by 185 of 1109 patients sampled within 24 hours of ICU admission (16.7%); 59 (5.3%) patients carried methicillin-resistant S. aureus (MRSA). Forty-four *S. aureus* (22 MRSA) acquisitions while on ICU were detected. Isolates were available for genetic analysis from 37 acquisitions. Whole-genome sequencing indicated that 7 of these 37 (18.9%) were transmissions from other colonized patients. Conventional methods (*spa* typing combined with overlapping patient stay) falsely identified 3 patient-to-patient transmissions (all MRSA) and failed to detect 2 acquisitions and 4 transmissions (2 MRSA).

***Conclusions.*** Only a minority of *S. aureus* acquisitions can be explained by patient-to-patient transmission. Whole-genome sequencing provides the resolution to disprove transmission events indicated by conventional methods and also to reveal otherwise unsuspected transmission events. Whole-genome sequencing should replace conventional methods for detection of nosocomial *S. aureus* transmission.

## Abstract (precis)

An assessment of Staphylococcus aureus acquisition among intensive care patients using serial sampling and whole-genome sequencing found less than a fifth of acquisitions resulted from patient-to-patient transmission. Whole-genome sequencing identified transmission more accurately than spa-typing and patient stay data.

**(See the Editorial Commentary by David and Daum on pages 619–21.)**

*Staphylococcus aureus* is a leading cause of healthcare-associated infection. Carriage usually precedes infection, and the risk of invasive disease is greatest immediately after acquisition of a new strain [1, 2]. Efforts to reduce *S. aureus* infections focus on preventing patient-to-patient transmission, including measures that target methicillin-resistant *S. aureus* (MRSA) such as screening and decolonization. In England, MRSA screening has been mandatory since 2006. Although MRSA bacteremia rates have fallen, rates of methicillin-sensitive *S. aureus* (MSSA) bacteremia remain high [3].

*Staphylococcus aureus* population structure is dominated by a few prevalent clones that account for most infections [4]. Strains of clonal complex (CC) 22 and 30 account for >95% of UK hospital MRSA cases. Conventional typing techniques lack the resolution necessary to differentiate closely related strains [5]. Reference laboratories commonly employ *spa* typing which has comparable resolution to multilocus sequence typing (MLST) and is more portable than pulsed-field gel electrophoresis (PFGE) [6, 7]. Although different *spa* types are usually genetically distant, a single base-pair change within the *spa* gene can produce 2 different but highly related *spa* types [8].

*Staphylococcus aureus* evolves primarily through point mutation, accumulating single-nucleotide variants (SNVs) over time [9]. Estimated mutation rates vary between 2.0 and 3.4 × 10^−6^ mutations per site per year [10–13]. This equates to 5.6–9.5 mutations per year over the whole genome or approximately 1 SNV difference every 5–10 weeks. Consequently, whole-genome sequencing reveals the genetic relatedness of isolates at far greater resolution than conventional techniques and implies a temporal relationship between isolates [14–17]. Costs and processing times will soon match those of conventional methods.

Here we report the first systematic evaluation of nosocomial patient-to-patient transmission of *S. aureus* using whole-genome sequencing. We analyzed weekly screening samples from all patients admitted to an adult intensive care unit (ICU). *Staphylococcus aureus* colonization status was determined by sampling on admission to ICU, followed by weekly sampling to detect acquisition. Patient-stay data and *spa* typing were used to identify acquisitions that could be attributed to patient-to-patient transmission using conventional typing and epidemiology. Whole-genome sequencing was then applied to assess the validity of conventional methods and determine more accurately the role of patients as sources of *S. aureus* in the ICU.

## METHODS

### Setting and Patients

The study was conducted on a 16-bed, adult ICU between 1 January 2010 and 28 February 2011 (14 months) in an acute teaching hospital (Brighton and Sussex University Hospitals) on the south coast of England. Routine practice was to screen all patients at admission and weekly thereafter. Screens consisted of nasal and perineal swabs plus additional samples for some patients from groin, sputum, urine, and wounds. From May 2010, patients were also screened at time of discharge. All patients received chlorhexidine washes, and MRSA-positive patients received nasal mupirocin. Anonymized demographic details and hospital stay data were collected from patient records.

### Definitions of Acquisition and Patient-to-Patient Transmission of *S. aureus*

*Staphylococcus aureus* acquisition was defined by a negative admission screen followed by a later positive screen; or a positive admission screen followed by later culture of a genetically different strain.

Patient-to-patient transmission on ICU was defined according to conventional criteria by acquisition of *S. aureus* with matching *spa* type and methicillin susceptibility of a strain cultured previously from a colonized patient with overlapping ICU stay; by whole-genome sequencing using SNV differences (irrespective of overlapping patient stay, to allow for indirect transmission via the environment or a vector), with a SNV difference of >40 used to exclude recent transmission [18].

### Microbiology

Swabs were plated directly onto chromogenic MRSAselect (Bio-Rad, Redmond, Washington) and Columbia CNA (Oxoid Ltd, Basingstoke, UK) agar. Plates were incubated at 35°C –37°C in air for 18 hours. Identification was confirmed with PROLEX Staph Xtra Latex Kit (Pro-Lab Diagnostics, Cheshire, UK) plus either RapiDEC-Staph (bioMérieux, Hampshire, UK) or Microflex series MALDI-TOF (Bruker Daltonics). Antibiotic susceptibilities were determined by disk diffusion [19]. Isolates were stored on ambient slopes and then transferred to Matrix 2D Barcoded Storage Tubes (ThermoFisher Scientific, Cheshire, UK) and stored at −80°C.

### *Spa* Typing

*Spa* typing was performed as described previously [20]. In brief, the X region of the *spa* gene was amplified by polymerase chain reaction (PCR) with primers 1095F (5′-AGACGATCCTTCGGTGAGC-3′) and 1517R (5′-GCTTTTGCAATGTCATTTACTG-3′) (Invitrogen), and PCR products were purified using Agencourt AMPure XP beads (Beckman Coulter, London, UK). Following sequencing, products were purified using Agencourt CleanSEQ beads (Beckman Coulter, London, UK). Sequencing was performed on an ABI 3730 DNA Analyzer (Applied Biosystems). The *spa* types were determined using Ridom StaphType (Ridom GmbH, Germany).

### Whole-Genome Sequencing

Cultures were incubated overnight on individual Columbia Blood Agar (Oxoid, Basingstoke, UK). DNA was purified from a 5-µL loop sweep of culture growth using QuickGene DNA tissue kits (Autogen) according to the manufacturer's instructions.

Whole-genome sequencing was performed on the Illumina HiSeq2000 using previously described protocols for bacterial library preparation and bioinformatics processing [14] in which SNVs in mapped nonrepetitive sites were identified by mapping to a CC30-specific reference (MRSA252 [21]). A minimum of at least 5 reads, with at least 1 in each direction, and a consensus of >75% were required to call a SNV, which was required to be homozygous under a diploid model. Across the sequenced genomes, a mean of 87% of the MRSA252 reference genome was called, and 95% of all reads generated mapped to the reference at a mean read depth of 126 (SD, 20). Maximum likelihood trees were estimated from mapped whole genomes using PhyML [22] under a Jukes-Cantor model.

Sequencing data were used to measure genomic diversity within and between hosts. To assess the diversity present within individuals over time and within sites, we measured the maximum pairwise genetic differences between isolates obtained from the same individual from the same site (eg, nose or groin) over time and between sites at the same time excluding acquisition isolates. To assess diversity between individuals, we measured the minimum pairwise genetic diversity between isolates obtained within 24 hours of admission to the ICU and any previous admission isolate.

### Ethics

This study was part of the Modernising Medical Microbiology consortium “Integrating Strain Typing and Database Technologies in Research Service” approved by the Berkshire Research Ethics Committee (reference 10/H0505/83) and the National Information Governance Board Ethics and Confidentiality Committee (reference ECC 8–05 (e)/2010).

## RESULTS

We investigated 1181 ICU patient stays involving 1065 patients. Median age at admission was 64 years (interquartile range [IQR], 49–76 years), 688 (59%) patients were male, and the median length of ICU stay was 2.6 days (IQR, 1.0–5.6 days).

### Carriage of *S. aureus* at ICU Admission

**Figure 1.**
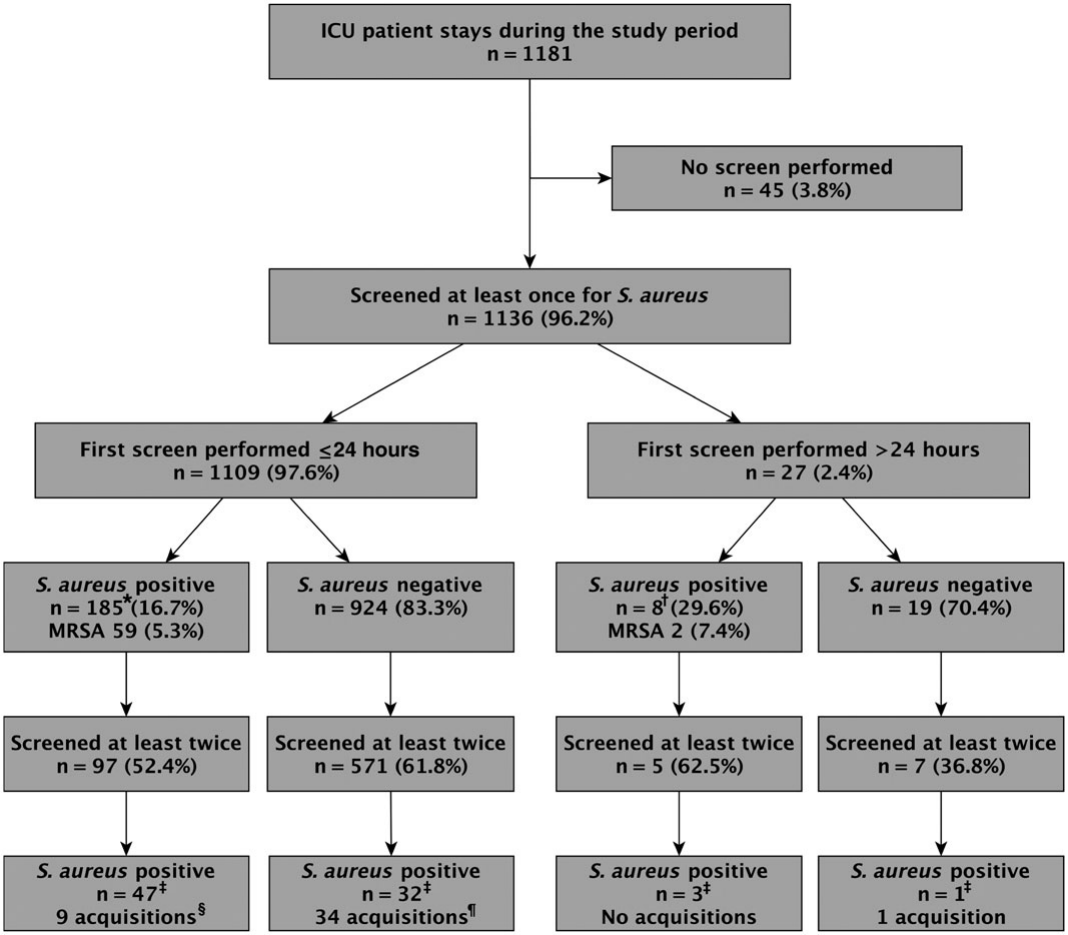
Sampling of patients involved in the study. Including nasal and extranasal samples and serial samples, *Staphylococcus aureus* was isolated 329 times as follows: 206 isolates (*), 8 isolates (†), 115 isolates (‡). Includes 1 patient who changed strains twice (§) and 1 patient who experienced 2 strain changes after acquiring *S. aureus* (¶). Abbreviations: ICU, intensive care unit; MRSA, methicillin-resistant *Staphylococcus aureus*.

A first screen for *S. aureus* carriage was conducted within 24 hours of admission to ICU in 1109 patients (93.9% of admissions). A total of 1104 (99.5%) had both nasal and extranasal swabs taken. One hundred eighty-five patients (16.7%) yielded *S. aureus*, and 59 (5.3%) MRSA (Figure 1). A further 27 patients had a first screen performed >24 hours after ICU admission.

### *Staphylococcus aureus* Acquisition on ICU

**Figure 2.**
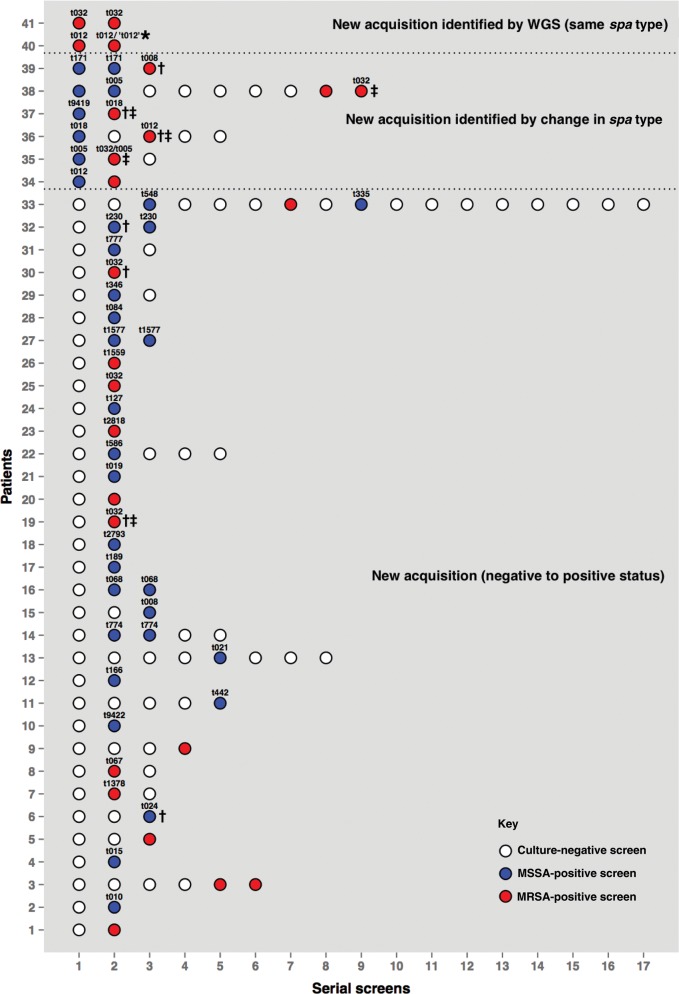
Sampling histories of 41 patients who acquired *Staphylococcus aureus*. Patient 33 experienced 3 separate acquisition events, and patient 35 experienced 2 acquisitions. *Routine *spa* typing assigned this isolate to t012, but whole-genome sequencing (WGS) revealed that it actually belongs to a different lineage. Patient-to-patient transmissions are identified as transmissions identified using WGS (†) and transmissions indicated by conventional criteria (‡; *spa* typing and overlapping stay). Abbreviations: MRSA, methicillin-resistant *Staphylococcus aureus*; MSSA, methicillin-sensitive *Staphylococcus aureus*; WGS, whole-genome sequencing.

Six hundred eighty patients had ≥2 samples taken to assess *S. aureus* carriage during their ICU stay (Figure 1). These identified 44 acquisitions, 22 of which were MRSA, in 41 patients as follows (Figure 2): 33 patients who were negative for *S. aureus* carriage on a first screen yielded *S. aureus* from a later screen (1 of these subsequently acquired 2 new *spa* types sequentially; hence, 35 acquisitions in this group). Additionally, 6 patients acquired new *spa* types (1 patient acquired 2 new *spa* types sequentially; hence, 7 acquisitions in this group). Furthermore, 2 acquisitions were detected solely by whole-genome sequencing. One patient, colonized with MRSA t032 at admission, acquired a genetically distinct isolate (222 SNVs) of the same *spa* type 2 days later. Another patient colonized with *spa* type t012 at admission subsequently acquired a genetically highly distinct (41 518 SNVs) strain. Routine *spa* typing had assigned this isolate to t012; whole-genome sequencing revealed that it actually belonged to a different lineage.

### *Staphylococcus aureus* Diversity Assessed by *spa* Typing

Including all samples from all patients, *S. aureus* was cultured from 329 samples. Isolates from 53 samples could not be retrieved from storage, and 1 failed *spa* typing (and whole-genome sequencing) leaving 275 isolates (Supplementary Table). Three *spa* types (t032 corresponding to CC22 [38.2%], t018 corresponding to CC30 [19.1%], and t012 corresponding to CC30 [10.3%]) accounted for 67.6% of all MRSA isolates. MSSA isolates were more diverse, with only 2 (t084 [6.8%] and t012 [5.8%]) comprising >5% of the total (Supplementary Figure 1).

### *Staphylococcus aureus* Transmission as Indicated by *spa* Typing and Overlapping Patient Stay

Seven acquisition isolates could not be retrieved for typing, leaving 37 of 44 evaluable acquisition events. Of these, 5 (14%) met conventional criteria for patient-to-patient transmission defined by overlapping stay with a patient carrying the same *spa* type. All 5 involved MRSA strains (Figure 2): patient number 37 (MRSA t018), patient number 36 (MRSA t012), and patient numbers 19, 35, and 38 (MRSA t032). All 3 t032 acquisitions occurred within 3 weeks of each other, suggesting a possible outbreak. Two other patients on the ICU at this time were found to carry MRSA t032; 1 was patient number 41, who acquired the strain 2 days before patient 35; the other was culture positive on primary screening and hence a possible donor to patients 19 and 38.

### *Staphylococcus aureus* Diversity Assessed by Whole-Genome Sequencing

**Figure 3.**
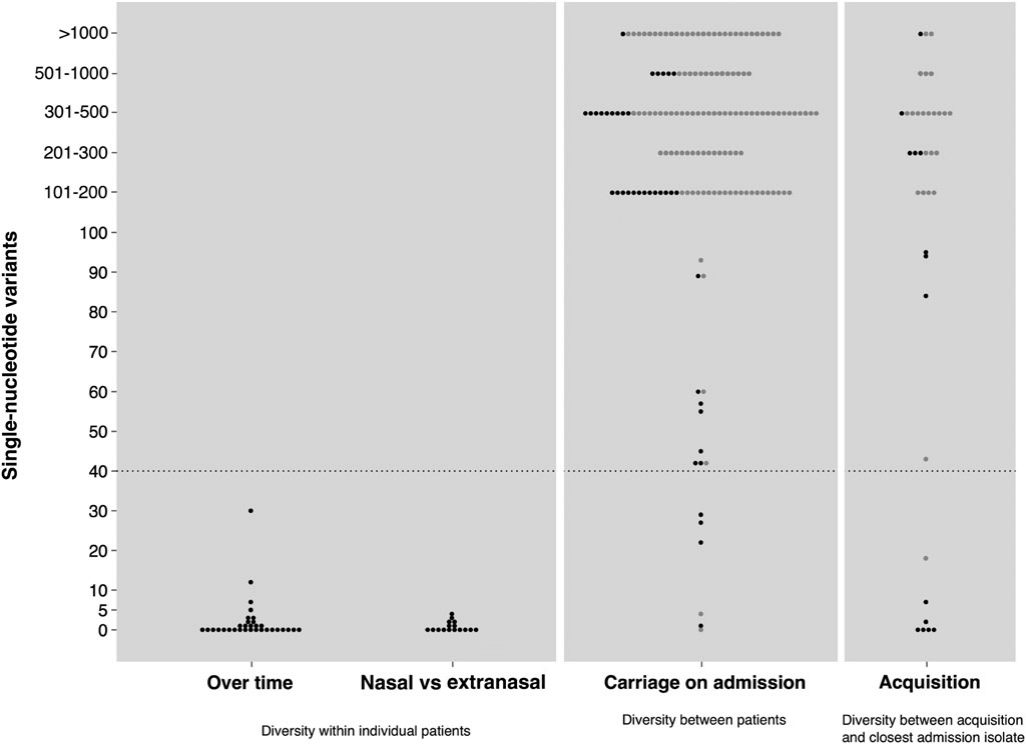
*Staphylococcus aureus* diversity shown by pairwise genetic distances in single-nucleotide variants: maximum diversity within individual patients over time and between nasal and extranasal sites; minimum diversity between different patients (for isolates cultured within 24 hours of intensive care unit admission); minimum diversity between new acquisitions and isolates cultured within 24 hours of admission. Each dot represents a pair of isolates and is shaded according to whether the pair has concordant (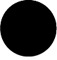) or discordant *spa* types (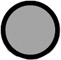).

Among 275 analyzed whole genomes, the diversity seen within and between hosts was markedly different (Figure 3). Within-host diversity, excluding acquisition isolates, was minimal either over time or between nasal and extranasal samples. Among 48 evaluable isolates (32 available to assess diversity over time and 16 to assess between-site diversity), 44 were within 4 SNVs of the most distant within-host isolate and all were within 40 SNVs, the maximum within-host diversity observed by Golubchik et al [18].

Among 160 admission isolates of *S. aureus* for which between-host diversity could be determined, 143 were >100 SNVs distant from the most similar isolate carried by another patient, and for 154 of 160 the minimum SNV difference was >40. Four admission isolates were highly related (<4 SNVs), suggesting a recent common source of acquisition; these patients originated from different clinical settings.

Evaluable acquisition isolates (n = 37) showed a bimodal distribution of minimum SNV differences compared to other *S. aureus* isolates sequenced, with 26 of 37 being >100 SNVs from the most closely related isolate sequenced in the study, 7 being <40 SNVs, and 5 being <4 SNVs.

### Transmission of *S. aureus* Assessed by Whole-Genome Sequencing

Whole-genome sequencing data allowed us to scrutinize the 5 instances of patient-to-patient transmission indicated by common *spa* type plus overlapping patient stay. The MRSA t018 isolate from patient 37 was genetically indistinguishable (0 SNVs) from the putative donor, confirming patient-to-patient transmission. The MRSA t012 isolate from patient 36 differed by 1100 SNVs from the putative donor isolate, disproving patient-to-patient transmission. However, the most closely related isolate was an MRSA t018 carried by a patient on the unit at the same time that differed by 18 SNVs. The “outbreak” identified by conventional criteria, in which patients 19, 35, 38, and 41 acquired MRSA t032, is illustrated in Figure 4*A* showing the ICU stays of all 17 patients from whom MRSA t032 was isolated in the study period (assigned letters A–Q). MRSA t032 isolates in the “outbreak” period from patients J and L/19 were highly related (2 SNVs), confirming patient-to-patient transmission, whereas isolates from the other 3 acquisitions (patients H/35, I/41, and K/38) were genetically distinct both from potential donors (J; 102–354 SNVs) and from each other (102–337 SNVs), disproving transmission.

**Figure 4.**
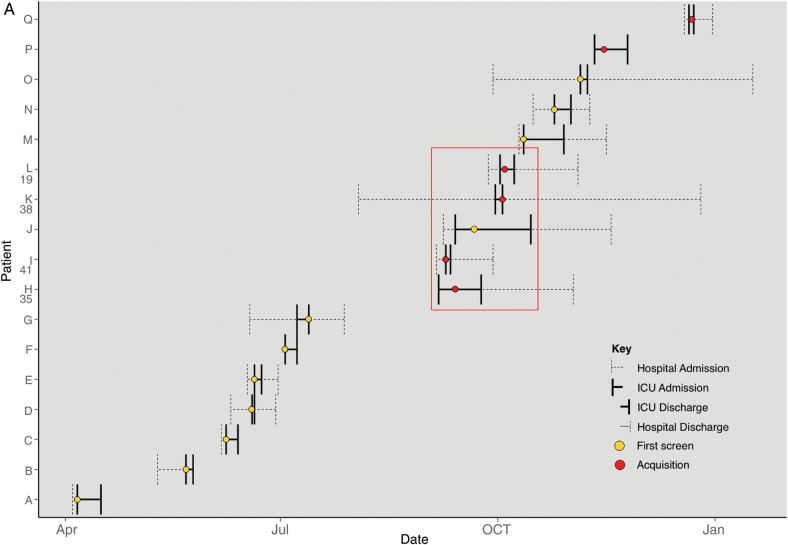
*A*, Intensive care unit stays of all 17 patients from whom methicillin-resistant *Staphylococcus aureus* (MRSA) t032 was isolated. Patients are labeled A–Q. The numbers 35, 41, 38, and 19 correspond with patients shown in Figure 2. The red box highlights a putative “outbreak” suggested by conventional criteria (same *spa* type plus overlapping stay). *B*, Maximum likelihood tree representing the genetic relatedness of MRSA t032 isolates in patients A–Q. The branch length reflects single-nucleotide variants (SNVs) identified between isolates (annotated). Nodes have been colored according to isolate type (red = acquisition isolate, yellow = carriage isolates, black = hypothetical node). Isolates sharing circles are genetically indistinguishable and touching nodes differ by 1 SNV. Twenty-six isolates were available from 17 patients, including 2 genetically distinct isolates from patient I (I1 and I2), 2 isolates from 7 patients (*), and 4 isolates from 1 patient (†). Abbreviation: ICU, intensive care unit.

**Figure 4:**
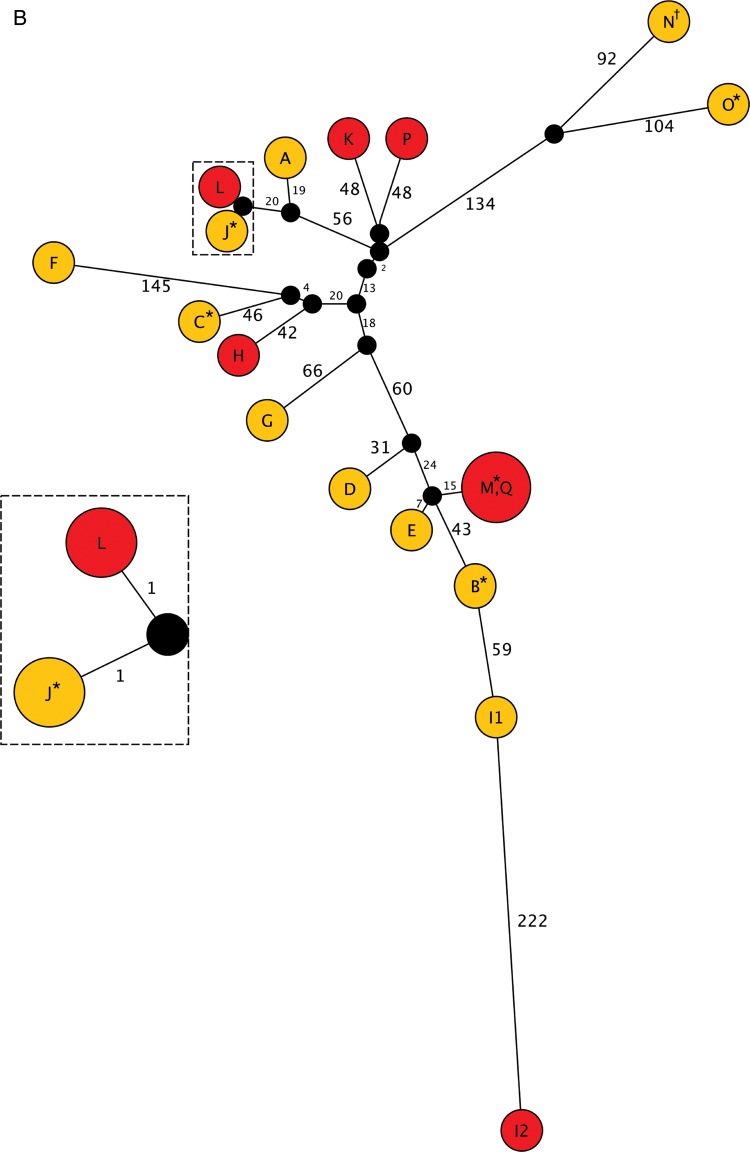
Continued.

### Whole-Genome Sequencing Reveals *S. aureus* Transmission Undetected by Conventional Methods

Two patients acquired MRSA t032 during the study period without sharing time on the ICU with any other patient known to be carrying this strain. These are patients 25 and 30 (patients P and Q) in Figure 4*A*. Analysis of the genetic relatedness of all MRSA t032 study isolates (Figure 4*B*) demonstrated that whereas patient P/25's isolate was 96 SNVs different from its nearest neighbor, the isolate from patient Q/30 was genetically indistinguishable (0 SNVs) from that carried by patient M, who was discharged 7 weeks earlier.

There were 2 other instances where patients acquired strains that were genetically indistinguishable (0 SNVs) from isolates identified earlier on the ICU despite the patient admissions being 18 and 34 days apart: 1 involved MRSA t008 (patient 39), the other MSSA t024 (patient 6), respectively.

One further acquisition involved highly related pairs of isolates. The MSSA t230 strain from patient 32 differed by 9 SNVs from an isolate of the same *spa* type colonizing a patient discharged from the ICU 107 days previously.

In total, whole-genome sequencing discounted 3 MRSA transmissions indicated by conventional criteria, confirmed 2 transmissions, and identified 5 additional transmissions (3 MRSA, 2 MSSA). These comprised 4 where isolates were genetically indistinguishable (3 with 0 SNVs) or closely related (9 SNVs) despite patients not having shared time on the ICU and 1 where *spa* types were discrepant despite isolates only differing by 18 SNVs and patients having shared time on the ICU.

## DISCUSSION

We undertook a systematic evaluation of nosocomial patient-to-patient transmission of *S. aureus* in an endemic setting in an unselected population of consecutive patients admitted to a typical ICU using whole-genome sequencing. Previous studies have demonstrated the value of whole-genome sequencing for investigation of *S. aureus* outbreaks [16, 17].

Among 680 patient stays evaluated with repeat sampling, we identified only 44 acquisitions, indicating that acquisition is uncommon among ICU patients at our hospital. We did not set out to study routes of acquisition prior to ICU admission, but it is noteworthy that among *S. aureus* isolates obtained within 24 hours of admission to ICU, a few were genetically highly related, suggesting acquisition from a common source prior to ICU admission.

Previous studies have measured *S. aureus* mutation rates at 5–10 SNVs per year [10–13], but to avoid underestimating the frequency of patient-to-patient transmission, we used a limit of >40 SNV differences to exclude recent transmission, given that SNV differences of up to 40 may be detected within an individual [18]. The observed within-host diversity indicates that this was a reasonable assumption, as all 7 acquisition isolates were <20 SNVs apart, 5 being <4 SNVs; only 1 acquisition isolate had the closest other isolate just outside the 40 SNV threshold.

Conventional investigations of transmission combine epidemiological information with typing of isolates. We show that use of a conventional approach to investigate transmission (*spa* typing and epidemiological association) falsely suggests transmission links between patients but also fails to identify transmission links, particularly where patient stays do not overlap, and that transmission might have occurred indirectly such as via healthcare workers or environmental contamination.

Efforts to prevent patient-to-patient transmission of *S. aureus* include measures such as hand hygiene and use of antiseptics, which target all *S. aureus*, plus measures such as MRSA screening and decolonization, which target MRSA [23, 24]. Rates of invasive MRSA infection have declined markedly in the United Kingdom, whereas rates of invasive MSSA infection have not. Although only a minority of *S. aureus* strains detected at admission to the ICU were MRSA, MRSA accounted for 50% of acquisitions and 5 of 7 patient-to-patient transmissions identified by whole-genome sequencing. These data suggest more frequent nosocomial transmission of MRSA. Patients were the source of only a minority of acquisitions, irrespective of methicillin susceptibility.

Our study has some limitations. It was performed in a single center and in the United Kingdom, a country with little reported community-based MRSA transmission; however, our findings should be generally applicable to ICUs where similar infection control measures are in place. Our analysis is based on carriage, not disease, isolates. Although patients with clinically significant *S. aureus* infections typically carry the same strain [2], it is possible that we missed some patient sources. We did not screen for throat or rectal carriage [25]. A proportion of isolates could not be retrieved for genetic analysis. This occurred throughout the study and is unlikely to have distorted our results. Our finding of related isolates in patients who did not share time on the ICU suggests that some acquisitions may have occurred outside the unit. Low-level *S. aureus* carriage may not be detected. Although 99.5% of our patients were sampled from ≥2 sites, our figure of 16.7% may underestimate the frequency of carriage at ICU admission. Previous studies report rates between 14% and 53% among hospitalized patients [26, 27], with higher rates (18%–46%) in high-dependency settings [28]. Although more sensitive sampling methods might have detected higher rates of carriage, it is likely that our patients were subject to more antistaphylococcal decolonization measures than patients recruited to these studies. Underestimation of *S. aureus* carriage on ICU would be expected to result in overestimation of the rate of *S. aureus* acquisition. However, the rate of acquisition we have observed is relatively low. Bloemendaal et al reported an acquisition rate of 14.2% in a study of 6 European ICUs [28]. Our fundamental observation, that only 18.9% of acquisitions can be attributed to transmission from other patients, cannot be explained by low sensitivity of the sampling method unless patients with false-negative screening results make a disproportionately high contribution to transmission. This seems unlikely.

A major strength of our study is that 94% of patients were sampled within 24 hours of admission, 99.5% of these at 2 sites, providing confidence in our conclusion that patients are not the major source of *S. aureus* acquisition in the ICU. Before the introduction of enhanced hand hygiene and universal antiseptic use, patient-to-patient transmission of *S. aureus* may have been more common. Our findings do not undermine the importance of current infection control practices; rather, they support their efficacy. They indicate that whole-genome sequencing can be used to assess the efficacy of infection control measures to prevent *S. aureus* transmission. Studies to evaluate other nosocomial sources of *S. aureus* (eg, the environment, healthcare workers, visitors, the food chain) are challenging. Substantial sample sets and clinical data and analysis would be required. Healthcare workers and hospital managers are anxious about identifying staff as potential sources of *S. aureus*. Nevertheless, our findings demonstrate that such studies are required and emphasize the value of whole-genome sequencing.

## Supplementary Data

Supplementary materials are available at *Clinical Infectious Diseases* online (http://cid.oxfordjournals.org/). Supplementary materials consist of data provided by the author that are published to benefit the reader. The posted materials are not copyedited. The contents of all supplementary data are the sole responsibility of the authors. Questions or messages regarding errors should be addressed to the author.

Supplementary Data
