# Supplementary material for: Whole-Genome Sequencing Shows That Patient-to-Patient Transmission Rarely Accounts for Acquisition of Staphylococcus aureus in an Intensive Care Unit
Source: Clin Infect Dis. 2013 Dec 12;58(5):609–18. doi: 10.1093/cid/cit807 (PMC3922217; doi:10.1093/cid/cit807)
Supplement: Supplementary Data [file supp_58_5_609__index.html]

Whole-Genome Sequencing Shows That Patient-to-Patient Transmission Rarely Accounts for Acquisition of Staphylococcus aureus in an Intensive Care Unit — Whole-Genome Sequencing Shows That Patient-to-Patient Transmission Rarely Accounts for Acquisition of Staphylococcus aureus in an Intensive Care Unit — Supplementary Data 

# Whole-Genome Sequencing Shows That Patient-to-Patient Transmission Rarely Accounts for Acquisition of *Staphylococcus aureus* in an Intensive Care Unit

## Supplementary Data

Supplementary Data

**Files in this Data Supplement:**

- Supplementary Data - Docx file
