# Supplementary material for: Whole-Genome Sequencing Shows That Patient-to-Patient Transmission Rarely Accounts for Acquisition of Staphylococcus aureus in an Intensive Care Unit
Source: Clin Infect Dis. 2013 Dec 12;58(5):609–18. doi: 10.1093/cid/cit807 (PMC3922217; doi:10.1093/cid/cit807)
Supplement: Supplementary Data [file supp_cit807_cit807supp.docx]

**Supplementary Table.** Patients screened and swabs processed within the study. * 1 isolate failed preparation for *spa*-typing and whole-genome sequencing.

| **Number of screens** | **Carriage phenotype** | **Total Admissions** | **Number of positive swabs** | **Number of isolates yielded** | **Isolates lost** |
| --- | --- | --- | --- | --- | --- |
| 1 | Negative (no serial screen) | 365 | 0 | 0 | - |
|  | Positive (no serial screen) | 91 | 111 | 89 | 22 |
| >1 | Negative to Negative | 545 | 0 | 0 | - |
|  | Negative to Positive | 33 | 44 | 38 | 6 |
|  | Positive to Negative | 52 | 55 | 46 | 9 |
|  | Positive to Positive       same *spa*-type       different *spa*-type | 44  7 (6 patients) | 102  17 | 89  14* | 13  3 |
|  |  | 1136 | 329 | 276* | 53 |

**Supplementary Figure.** Distribution of *spa*-types among isolates identified in the study. Number of isolates of each *spa*-type is given and proportion of pie denotes MRSA (red) and MSSA (blue).
